# Speckle-Tracking Global Longitudinal and Regional Strain Analysis in Neonates with Coarctation of Aorta: A Case-Control Study

**DOI:** 10.3390/jcm10194579

**Published:** 2021-10-02

**Authors:** Liliana Gozar, Claudiu Mărginean, Andreea Cerghit Paler, Dorottya Gabor-Miklosi, Daniela Toma, Mihaela Iancu, Rodica Togănel, Amalia Făgărășan

**Affiliations:** 1 Emergency Institute of Cardiovascular Diseases and Transplantation, 540139 Târgu-Mureș, Romania; liliana.gozar@umfst.ro (L.G.); palerandreea@yahoo.com (A.C.P.); annadorka@yahoo.com (D.G.-M.); daniela.toma@umfst.ro (D.T.); rodica.toganel@umfst.ro (R.T.); amalia.fagarasan@umfst.ro (A.F.); 2 Department of Pediatrics, George Emil Palade University of Medicine, Pharmacy, Science and Technology of Târgu-Mureș, 540139 Târgu-Mureș, Romania; 3 Department of Obstetrics and Gynecology, George Emil Palade University of Medicine, Pharmacy, Science and Technology of Târgu-Mureș, 540139 Târgu-Mureș, Romania; marginean.claudiu@umfst.ro; 4 Department of Medical Informatics and Biostatistics, Faculty of Medicine, Iuliu Hațieganu University of Medicine and Pharmacy Cluj-Napoca, 400349 Cluj-Napoca, Romania

**Keywords:** coarctation of aorta, speckle-tracking, myocardial strain, neonate

## Abstract

Our objectives are to compare speckle-tracking peak global longitudinal (pGLS) and regional strain values in neonates with coarctation of aorta (CoA) and control groups. Echocardiographic parameters measured by speckle-tracking were studied in a retrospective single-center study. A comparison of pGLS and segmental deformation between neonates with CoA and control group was performed using a three-way mixed ANOVA model. There was a significant difference in the means of segmental strain values between CoA and control group at the apical (*p* = 0.018) and basal segments (*p* = 0.031) of the interventricular septum and at the apical segment (*p* = 0.026) of the left ventricle (LV). After correcting for multiple comparisons, the results had a tendency toward statistical significance (adjusted-*p* < 0.10). There was significant difference in the mean values of pGLS [F(1, 39) = 7.61, *p* = 0.009, adjusted *p* = 0.018] between the studied groups. The results of ROC analysis showed that a cut-off value of −16.60% for pGLS provided an estimated sensitivity of 92.31% (95% CI: [63.97, 99.81]) and 71.43% specificity (95% CI: [51.33, 86.78]) for the diagnosis of CoA in neonates (AUC = 0.794, 95% CI: [0.66, 0.93]). pGLS can be regarded as a feasible and reproducible parameter reflecting LV dysfunction in newborns with CoA when compared to newborns with a false-positive diagnosis.

## 1. Introduction

Neonatal aortic coarctation (CoA), defined as narrowing of the isthmic region of the aorta, is one of the ductal-dependent cardiac malformations commonly encountered in pediatric pathology, with an incidence of 5–8% [1]. Obstruction of the aortic arch becomes manifest due to the constriction of the arterial duct, more precisely in the first two weeks of life.

CoA in the newborn has significantly contributed to neonatal mortality and morbidity. Despite clinical protocols based on saturation determination and echocardiographic examination, a significant number of newborns with this pathology are diagnosed only a few days after discharge from the maternity ward with signs of critical heart failure and cardiogenic shock, due to the narrowing of the arterial duct [2].

Prenatal diagnosis has made important contributions in improving the postnatal diagnosis rate for newborns with CoA, despite the fact that due to fetal circulation this pathology does not manifest before birth [3]. Ventricular disproportion, although an echocardiographic aspect with reduced sensitivity and specificity, resulting in a significant number of false-positive results, may be the first sign to raise the suspicion for the diagnosis. Several other morphological aspects, such as hypoplasia of the ascending aorta and aortic arch, increase the sensitivity of the diagnosis [4,5,6,7]. Establishing the diagnosis remains difficult even postnatally, as long as arterial ductal patency and the ventricular disproportion are maintained. In the case of a newborn with this diagnostic suspicion, careful clinical monitoring and repeated echocardiographic evaluations are required until the confirmation or exclusion of this diagnosis. The transition from fetal to neonatal circulation is accompanied by changes in right ventricular afterload by the physiological decrease in the pulmonary vascular resistance. Additionally, ductal-dependent aortic coarctation is associated with modifications in the afterload for both ventricles. In these hemodynamic conditions, assessment of ventricular function may play an important role. Changes in ventricular function can be most often subtle and undetected by a standard echocardiographic evaluation. Evaluation of myocardial function by new echocardiographic methods such as speckle-tracking must find its place and role in the diagnosis and hemodynamic evaluation of fetuses and newborns with this pathology. Longitudinal myocardial strain and strain rate is the way in which the longitudinal deformation of the ventricles is quantified following an applied force [8,9]. To date, the diagnosis of a ductal-dependent CoA is based on morphological echocardiographic aspects, which in many cases may become evident only after the restriction of the arterial duct. The diagnostic algorithm lacks parameters for the evaluation of the ventricular function, and classical echocardiography is relatively limited.

In light of the above mentioned, we aimed to establish the usefulness of the speckle-tracking method in newborns with intrauterine suspicion of CoA and we outlined the following objectives: (i) to compare the speckle-tracking peak global longitudinal and regional strain in neonates with CoA and control groups, (ii) to assess the diagnostic performance of echocardiographic parameters for which differences were obtained between groups, and (iii) to evaluate the reproducibility of speckle-tracking echocardiographic measurements.

## 2. Materials and Methods

This is a retrospective single-center study conducted at the Children’s Cardiology Clinic, from the Institute of Cardiovascular Diseases and Transplantation, Târgu-Mureș, for a period of 3 years, from January 2018 until December 2020. All newborns, in which the suspicion of aortic coarctation had arisen in the last trimester of pregnancy, were included in this study. All patients were examined echocardiographically within the first 24 h postnatal period, then they were kept under clinical surveillance, and echocardiographic reevaluations were made daily until confirmation or exclusion of the diagnosis. The exclusion of the diagnosis could not be made in either of the cases within the first 24 h.

The inclusion criteria were the following: newborns examined echocardiographically in the first 24 h at the Children’s Cardiology Clinic who had intrauterine suspicion of CoA diagnosis based on ventricular disproportion, particularly anatomy of the aortic arch, with suboptimal dimensions and wide arterial duct; existence in the database of the first echocardiographic evaluation containing a 2D four-chamber view, recorded with a minimum frequency of 60 Hz.

The exclusion criteria were as follows: a gestational age of less than 36 weeks, low birth weight for gestational age, other associated heart malformations (ventricular septal defect, aortic stenosis, mitral stenosis, left ventricular hypoplasia, Shone syndrome, transposition of the great arteries), and systolic dysfunction with ejection fraction under 50%, maternal pathology (gestational diabetes, hypertension).

**Figure 1:**
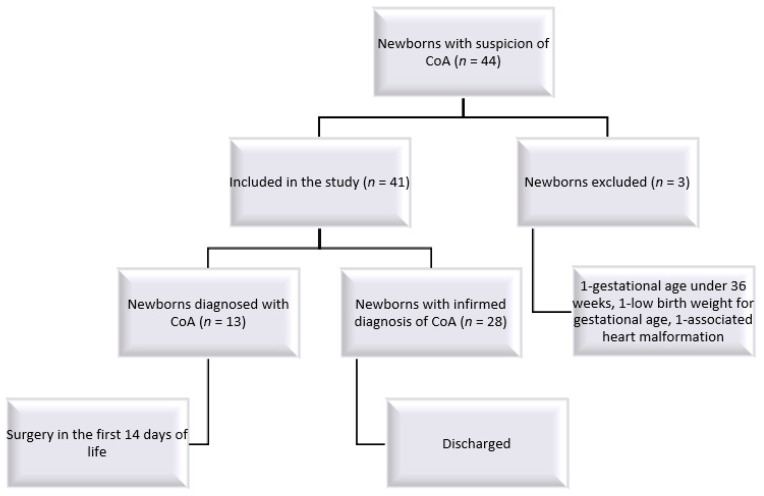
Study flow chart. CoA—aortic coarctation.

All echocardiographies were performed with an Epiq echocardiograph. The images of the newborns included in the study (Figure 1) were reviewed by an observer who saved the four-chamber views of the subjects in Digital Imaging and Communication in Medicine (DICOM) format. The recorded images were analyzed through speckle-tracking by two other observers who did not know which of the selected images belonged to subjects with CoA.

The control group was represented by newborns in whom aortic coarctation was not confirmed.

### 2.1. Speckle-Tracking Analysis

**Figure 2:**
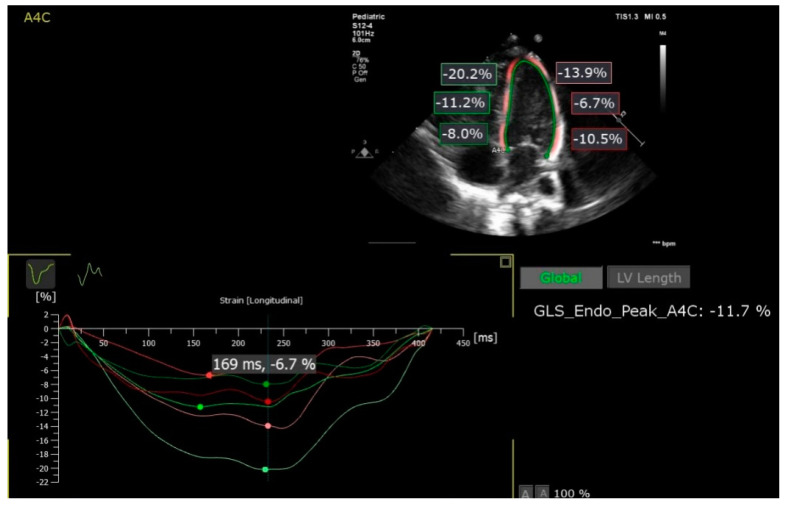
Left ventricle speckle tracking analysis; GLS_Endo_Peak_A4C (denoted by LV pGLS in our study)—peak global longitudinal strain of the left ventricle from apical 4-chamber view (autostrain LV, PhlipsQlab 13).

The selected 2D images were analyzed offline using a PhilipsQlab 13 software with autostrain left ventricle (LV) and autostrain right ventricle (RV) functions (Figure 2).

The cardiac cycle was defined in M mode by the movement (closing and opening, respectively) of the mitral valve generated automatically by the software, so that the analysis could be made on an entire cardiac cycle. Three points were marked at the level of the endocardium (base of the septum, lateral and apical). The edge of the endocardium was drawn automatically, then verified and corrected manually. The peak global longitudinal strain (pGLS) and segmental deformation were measured, the interventricular septum and the walls being divided into 3 segments (Table 1). The inter-observer variability of speckle-tracking analysis was evaluated by performing speckle-tracking analysis in all cases included in this study.

### 2.2. Ethics

The study was approved by the ethics committees of the Institute of Cardiovascular Diseases and Transplantation and George Emil Palade University of Medicine and Pharmacy Targu-Mures 1276/25.02.2021.

### 2.3. Statistical Analysis

Demographic and echocardiographic data were described as arithmetic mean ± SD (standard deviation) or median with interquartile interval (Q_1_, Q_3_) for quantitative variables, or as absolute (relative frequency) for qualitative variables. Comparison between neonates with CoA and control group concerning speckle-tracking global longitudinal and regional strain was performed after the evaluation of univariate and multivariate (where appropriate) normality by univariate statistics, QQplots, univariate and multivariate Shapiro tests. The comparison between two groups concerning demographic characteristics was performed using Student’s *t*-test, Mann–Whitney test, or Chi-square tests. Because we were interested in comparing CoA and control groups concerning regional strain values measured by speckle-tracking echocardiography, we performed a three-way mixed ANOVA. In the mixed ANOVA design, regional strain as an outcome variable was modeled as a function of the cardiac wall (LV lateral wall, RV free wall, interventricular septum) and cardiac segment (apical, medial, basal). We also tested the 3-way and 2-way interaction between the 3 independent factors (group, wall, and cardiac segment). In the case of significant interaction term, the analysis was followed up by pairwise analysis using Student’s *t*-test with a post hoc adjustment of *p*-values by the Benjamini–Hochberg procedure.

The difference between CoA and control groups regarding longitudinal strain values measured by speckle-tracking echocardiography was assessed by multivariate analysis of variance (MANOVA). The LV pGLS measured on the LV, longitudinal strain measured on the free wall of right ventricle, and longitudinal strain on the RV in 4-chamber view were considered as three correlated, dependent variables. Because of the presence of multicollinearity between RV free walland RV4C, the former was excluded from MANOVA analysis. After ascertainment of the multivariate normality and sphericity condition (by Box’s M-test for Homogeneity of Covariance Matrices), we performed a MANOVA analysis. In order to identify the source of differences inmeans of longitudinal strain values between groups, a one-way ANOVA was done as post hoc analysis with a post hoc adjustment of *p*-values by the Benjamini–Hochberg procedure.

Diagnostic performance of significant echocardiographic parameters was evaluated by the area under receiver operating characteristic (AUC) and associated 95% confidence interval.

Interrater reliability analysis was performed using intraclass correlation coefficients (ICC) and 95% confidence interval. A two-way mixed-effects model was used to determine consistency of cardiac speckle-tracking measurements between the 2 raters. The level of reliability was interpreted using the confidence interval for ICC: poor reliability (95% CI contained values lower than 0.5), moderate reliability (95% CI contained values between 0.50 and 0.75), good reliability (95% CI contained values between 0.75 and 0.90), and excellent reliability (95% CI contained values greater than 0.90) [10].

Absolute Agreement between 2 assays of speckle-tracking echocardiography parameters was evaluated by Bland–Altman analysis. The mean differences (bias) were determined as a measure of accuracy, while 95% CI for bias was estimated as a measure of precision (95% CI: Lower limit = mean differences − 1.96 SE_diff_, Upper limit = mean difference + 1.96 SE_diff_, where SE_diff_ was the standard error of differences between the two raters regarding the evaluated cardiac parameters). If the 95% confidence interval of the bias contained the value 0, there was no statistical evidence for systematic bias between the measurements of the 2 raters. The 95% limits of agreement (lower LoA and upper LoA) were also calculated in order to quantify the total bias, the lower LoA = bias − 1.96 SD_diff_ and upper LoA = bias + 1.96 SD_diff_ where SD_diff_ was the standard deviation of differences between the measurements of the2 raters. The paired Student’s *t*-test was also used to test if the mean differences differed significantly from zero for each studied cardiac parameter.

All applied statistical tests were two-sided with a significance level (α) chosen at 0.05.

All statistical analyses were performed using the R software version 4.04 (R Foundation for Statistical Computing, Vienna, Austria).

## 3. Results

During the above-mentioned period, there were 44 newborns with the suspicion of CoA, but only 41 were eligible for inclusion in the study: 13 newborns in whom the diagnosis of CoA was confirmed postnatally and required surgery in the first 14 days of life and 28 newborns, respectively, for whom the suspicion of the diagnosis was not confirmed and were discharged from the neonatal ward as healthy newborns. All patients from the latter group went on to be reevaluated clinically and by echocardiography at 1, and 3 months, with none of them developing coarctation of the aorta.

### 3.1. Description of the Studied Groups

The baseline characteristics of 13 neonates with CoA (CoA group) and a group of 28 healthy neonates (Control group) were described in Table 2. No significant difference in age distribution was observed between the two groups (Mann–Whitney test, *p* = 0.362). Similar results were obtained concerning sex distribution (Chi-square test, *p* = 1.00) and gestational age (Student’s *t*-test, *p* = 0.056), but there was a significant difference in mean weight between the two groups (Student’s *t*-test, *p* = 0.0003).

There was no significant difference in frequencies of LV (Fisher’s exact test, *p* = 0.541) or RV asynchronism (Fisher’s exact test, *p* = 1.00).

The frame rate for the echocardiographic exam was kept between 61–101 Hz, with a median frame rate of 90 Hz and no significant differences between neonates with CoA and controls (Student’s *t*-test, *p* = 0.440)

### 3.2. Comparison of Segmental Strain Values between CoA and Control Groups

**Figure 3:**
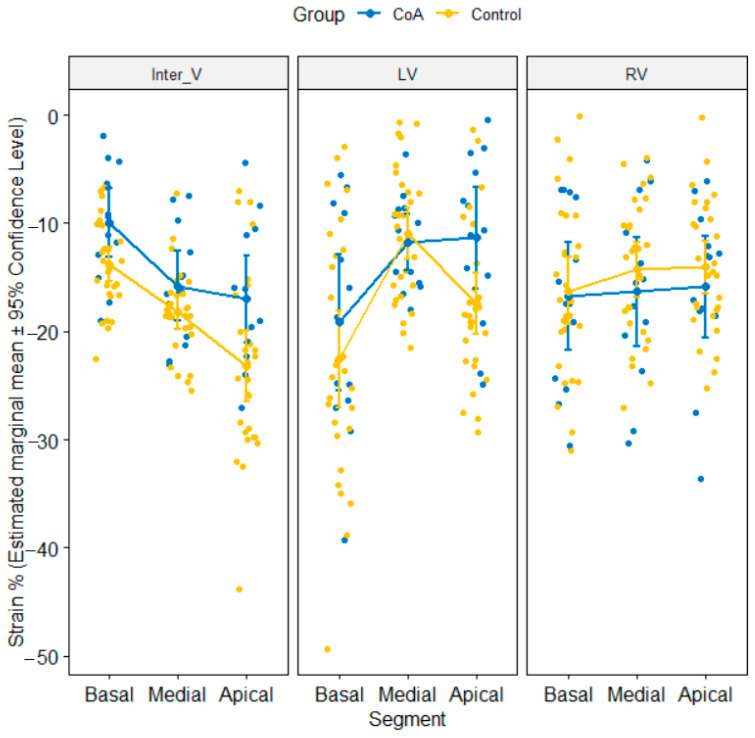
Estimated marginal mean values with the respective 95% confidence intervals of the mean strain values by Group stratified by the three walls and three segments per wall. CoA—aortic coarctation, Inter V—interventricular septum, LV—left ventricle, RV—right ventricle.

We performed a 3-way mixed ANOVA on the strain values (%) with Groups (CoA versus Control) as between factor, wall (LV free wall, RV free wall, interventricular septum) and segment (apical, medial, basal) as within factors. There was no statistically significant main effect of group [F(1, 39) = 1.68, *p* = 0.202] and main effect of wall [Greenhouse–Geissercorrection: F(1.86, 72.56) = 0.39, *p* = 0.661]. There was a tendency toward statistical significance for main effect of segment on strain values [Greenhouse–Geissercorrection: F(1.53, 59.57) = 2.58, *p* = 0.098]. There was no significant 3-way interaction between group, wall, and segment (*p* = 0.377), but the group-by-wall and wall-by-segment were significant interaction terms on strain values (*p* = 0.017). The results of pairwise tests showed the that mean strain value was significantly different in the CoA group when compared to thecontrol at the apical and basal segments of inter-ventricular septum, respectively, at the apical segment of LV (Table 3, Figure 3). After correcting for multiple comparisons, the results had a tendency toward statistical significance (*p* < 0.10).

We noticed that mean value of strain values measured on the left ventricle in the CoA group was equal to −14.08 ± 4.03 versus −16.99 ± 5.46 in control group while mean value of strain values on the right ventricle in CoA group was −16.34 ± 7.73 versus −14.87 ± 6.64 in controls

### 3.3. Comparison of Longitudinal Strain Values between CoA and Control Groups

A one-way MANOVA was performed to determine if the LV pGLS values measured on LV and longitudinal strain values measured on the RV4C were significantly different between the studied groups. There was a statistically significant difference between the CoA and control groups on the combined dependent variables (LV pGLS and RV4C), F(2, 38) = 5.98, *p* = 0.0055. The results of post hoc analysis obtained by one-way ANOVA showed that there was a statistically significant difference in mean values of pGLS [F(1, 39) = 7.61, *p* = 0.009, adjusted *p*_BH_ = 0.018] between CoA and control group (mean ± SD: −13.72 ± 3.88 versus −17.29 ± 3.83 for CoA and control group). There was no statistical evidence for significant difference in the mean value of RV4C between CoA and control groups (adjusted p_BH_ = 0.793, mean ± SD: −13.97 ± 5.99 versus −13.51 ± 4.68 for CoA and control group).

### 3.4. ROC (Receiver-Operating Characteristic Curve) Analysis

The LV pGLS with a cut-off value of −16.60% provided a value of 92.31%, 95% CI: [63.97, 99.81] sensitivity and 71.43% specificity, 95% CI: [51.33, 86.78] for the diagnosis of CoA in neonates (AUC = 0.794, 95% CI: [0.66, 0.93]).

### 3.5. Reliability Analysis of Speckle-Tracking Parameters in the Whole Sample (n = 41)

Biventricular as well as segmental strain values were measured by two raters. The level of reliability was moderate-to-good for basal and apical segments of the LV lateral wall, for medial and apical segments of the interventricular septum, and for LV pGLS measurements, while for apical, basal, medial segments of RV, and longitudinal measurements on the free wall of RV and RV4C, we found a good to excellent reliability (Table 4). The medial segment of LV and basal segment of the interventricular septum showed a point estimate of ICC, which denoted a moderate reliability, but taking into account the confidence interval limits for ICC, the level of reliability for the true ICC was regarded as poor to moderate.

The Bland–Altman analysis (Figures S1–S3) showed that there was an absolute agreement between the 2 raters concerning all cardiac speckle-tracking parameters except for apical segment of the interventricular septum (Inter V Apical: bias = 2.61, 95% CI: [0.99, 4.22]).

## 4. Discussion

The present study aimed to evaluate the postnatal ventricular parameters in neonates with CoA and control groups and to quantify the performance of echocardiographic parameters in the diagnosis of CoA. It is a comparative study that followed the differences in global and segmental ventricular deformation in newborns with CoA. To our knowledge, this is the first study from Romania and within the first few published in the international literature, performed on a group of newborns with intrauterine suspicion of aortic CoA, which aimed to compare the cases in which CoA was confirmed to the ones where it was not. All newborns included in the study showed significant ventricular disproportion and suboptimal size of the aortic arch or disproportion of the large vessels at the intrauterine evaluation. However, the diagnosis of aortic coarctation was confirmed in only 13 newborns. It is known that ventricular disproportion has moderate sensitivity and low specificity in the diagnosis of aortic coarctation, making other echocardiographic parameters necessary in order to reduce the false-positive diagnostic rates. It should also be mentioned that among fetuses with the suspicion of aortic coarctation, the percentage of false-positive cases is higher in the last trimester, when compared to fetuses in whom the suspicion arises at a younger gestational age [7].

The feasibility of the method of evaluating myocardial deformation by speckle-tracking in newborns is documented in several previous studies [9,11,12,13]. This method allows the determination of a global functional parameter, LV pGLS, as well as segmental evaluation, an important analysis considering the multitude of anatomical and functional factors involved in obtaining the beating flow.

The LV has a smooth ventricular cavity and myocardium formed by longitudinal and circumferential fibers, the deformation during systole being formed by longitudinal and circumferential shortening and radial thickening [14,15]. A study published by Sequela et al. was the first to study the feasibility of the speckle-tracking method in newborns with CoA, in the group of 6 newborns with CoA, the LV pGLS value being significantly lower when compared to a group of healthy newborns [16]. In our study, the results were similar, the overall longitudinal deformation of the LV pGLS, had a lower value in newborns with CoA (−13.72 ± 3.88) than in controls (−17.29 ± 3.83).

In the neonatal period, the shape and kinetics of the interventricular septum are indicators of the hemodynamic and pressure changes characteristic of this period [17,18]. In the present study, the deformation of the interventricular septum was analyzed in the three segments: basal, median and apical. We noticed the altered values of strain and strain rate on the apical and basal segments in newborns with CoA when compared to newborns in which this diagnosis was not confirmed, the observed difference having a tendency towards statistical significance. In this study, we compared two groups of newborns, one group with confirmed CoA patients and the second consisting of newborns in which CoA was not confirmed within a few days, but in whom the right cavities were more dilated in the first 24 h when the first echocardiography was performed.

The RV can be characterized by thin walls and a cavity with a more complex geometry and architecture in comparison to the LV. The contraction of the longitudinal fibers (located in the depth) determine the dominant deformation, which significantly contributes to the beating flow [14,15]. The high longitudinal shortening capacity of the RV may explain why there are no significant differences between the longitudinal strain values at the level of the segments that make up the free wall of the RV.

We also studied the synchronism in the contraction of the ventricular segments, both for the left ventricle and for the right ventricle, without obtaining statistically significant changes between the two groups.

CoA is a widely debated topic in the scientific literature from the recent years, being a clinical entity with a significant impact on neonatal mortality and morbidity. Prenatal diagnosis of CoA is of major importance in reducing neonatal mortality [3]. Highlighting ventricular disproportion is the prenatal echocardiographic element that most frequently raises the suspicion of CoA. The association between ventricular disproportion diagnosed in the third trimester and postnatal confirmation of coarctation of the aorta is reduced. The presence of disproportion between the great arteries and aortic arch hypoplasia in association with ventricular disproportion increases the sensitivity and specificity of the diagnosis [5,19,20]. In a meta-analysis recently published by Familiari et al., the authors, which summarized an evidence from 12 studies, concluded that a multiparametric diagnostic model can increase the detection rate of this condition [21].

The usefulness of quantification of myocardial deformation by the speckle-tracking method in assessing ventricular function was demonstrated not only in healthy fetuses [22], but in fetuses with heart defects, as well [23,24].

The study published by Miranda demonstrates the usefulness of the speckle-tracking method for analyzing myocardial function in fetuses with CoA. In the mentioned study the variability of results between observers is possibly due to the analysis of images with suboptimal frame rates. Fetuses with CoA have low LV pGLS and strain rates in comparison to the control group, and there are also regional differences at the level of the LV free wall, respectively, however, with no significant differences in the free wall of the RV [25]. In our study, we also analyzed the inter-rater variability and reliability analysis and we found a moderate to excellent point estimation of inter-rater reliability coefficient.

DeVore proposes several 2D echocardiographic parameters that evaluate the ventricular size, shape and function, and the global and segmental speckle-tracking analysis, respectively. The use of logistic regression analysis demonstrates the possibility of making the diagnosis between fetuses with CoA, when compared to fetuses with false-positive diagnosis [26]. As in the fetal period, the postnatal clinical diagnosis of CoA in the presence of the persistent arterial duct is often difficult to establish. In the clinical practice, the exclusion of CoA in newborns with fetal suspicion is extremely difficult. The relationship between left and right sided structures cannot distinguish false-positive and false-negative cases. Furthermore, the arterial duct improves the flow in the descending aorta and causes overestimation of the diameter of the aortic isthmus [16,27]. In our study, we found that an optimal cut-off value of −16.6% for overall longitudinal deformation of the LV pGLS demonstrated a good ability in discriminating between neonates with CoA and those without CoA, having a sensitivity, specificity, and a positive likelihood ratio value of 92.31%, 71.43%, and 3.23 with an area under the curve of 0.79.

The main limitation of this study is the retrospective nature of the data and the lack of a group of healthy newborns, examined on their first day of life in order to establish the optimal cut-off value likely to discriminate between healthy neonates and those with CoA. Another limitation is related to the small sample size, although the prevalence of CoA is known to be low. Although strain rates would have been more sensitive, they were not computed due to software limitation and the failure to do so represents a limitation of our study. Because of retrospective design of the study only A4C view images were available for evaluation. Given their small ages, obtaining transverse images to compute circumferential strain would be difficult in neonates. In addition, although we highlighted the potential utility of the LV pGLS to discriminate neonates with CoA from newborns with false-positive diagnosis in the presence of a persistent arterial duct, more prospective studies with a larger number of neonates with CoA would be needed to establish if LV pGLS can be considered useful in the diagnosis of ductal-dependent aortic coarctation or to characterize the myocardial function and predict the CoA risk in neonates as an independent factor adjusting for other potential covariates.

## 5. Conclusions

The speckle-tracking analysis is a feasible and reproducible method able to discriminate neonates with CoA from newborns with false-positive diagnosis in the presence of a persistent arterial duct. The speckle-tracking peak global longitudinal strain (LV pGLS) can be considered useful in the diagnosis of ductal-dependent aortic coarctation in neonates with suspected prenatal CoA. The study of the deformation of segments of the interventricular septum could represent an area of interest for a prospective study, as parameters seem to have diagnostic value.

## Figures and Tables

**Table 1:** Description of measured Speckle-tracking Variables.

Measured Variable	Abbreviation	Description
Left ventricle lateral wall basal	LV Basal	Strain measured on the basal portion of the left ventricle lateral wall
Left ventricle lateral wall medial	LV Medial	Strain measured on the medial portion of the left ventricle lateral wall
Left ventricle lateral wall apical	LV Apical	Strain measured on the apical portion of the left ventricle lateral wall
Inter-ventricular septum basal	Inter V Basal	Strain measured on the basal portion of the inter-ventricular septum
Inter-ventricular septum medial	Inter V Medial	Strain measured on the medial portion of the inter-ventricular septum
Inter-ventricular septum apical	Inter V Apical	Strain measured on the apical portion of the inter-ventricular septum
Left ventricle peak global strain	LV pGLS	Peak Global longitudinal strain of the left ventricle
Right ventricle lateral wall basal	RV Basal	Strain measured on the basal portion of the right ventricle lateral wall
Right ventricle lateral wall medial	RV Medial	Strain measured on the medial portion of the right ventricle lateral wall
Right ventricle lateral wall apical	RV Apical	Strain measured on the apical portion of the right ventricle lateral wall
Right ventricle free wall	RV free wall	Longitudinal strain of the right ventricle free wall
Right ventricle global4-chamber longitudinal strain	RV4C	Global longitudinal strain of the right ventricle from 4-chamber view

**Table 2:** Baseline characteristics of control and CoA groups.

	Control Group (*n*_1_ = 28)	CoA Group (*n*_2_ = 13)	*p*-Value
Gestational Age (weeks), mean ± SD	39.18 ± 0.98	39.77 ± 0.83	0.056
Age (days), median (Q1, Q3)	2 (2.00, 3.25)	1 (1.00, 6.00)	0.362
Sex (male), *n* (%)	15 (53.57)	7 (53.85)	1.000
Weight (g), mean ± SD	3474 ± 257.85	3725 ± 145.01	0.0003 *
Systolic blood pressure, mean ± SD	83.8 ± 2.6	83.8 ± 2.7	0.954
Heart rate, mean ± SD	135 ± 7.9	134.6 ± 8.2	0.743
LV asynchronism, *n* (%)			0.541
Synchronism	9 (32.14)	4 (30.77)	
1 asynchronous segments	6 (21.43)	5 (38.46)	
2 asynchronous segments	6 (21.43)	3 (23.08)	
≥3 asynchronous segments	7 (25.00)	1 (7.69)	
RV asynchronism, *n* (%)			1.000
Synchronism	22 (78.57)	10 (76.92)	
1 asynchronous segment	6 (21.43)	3 (23.08)	

Mean = aritmetic mean; SD = standard deviation; Q1 = first quartile; Q3 = third quartile; LV asynchronism (1 segment asynchronous versus synchronous); RV asynchronism ((1 segment asynchronous versus synchronous); *n* = number of subjects; *p*-values obtained from Student’s *t*-test, Mann–Whitney test or Fisher’s exact test; * statistical significant *p*-values (*p* < 0.05), LV—left ventricle, RV—right ventricle.

**Table 3:** Results of pairwise comparisons of segmental strain values between CoA and control groups.

Wall	Cardiac Segment	Group	Mean (SD)	*p*-Value	Adjusted p_BH_-Value
Inter V	Apical	CoA	−17.02 (6.70)	0.018	0.093
		Control	−23.19 (8.54)		
Inter V	Basal	CoA	−9.93 (5.28)	0.031	0.093
		Control	−13.81 (4.15)		
Inter V	Medial	CoA	−15.80 (5.32)	0.157	0.353
		Control	−18.25 (4.08)		
LV	Apical	CoA	−11.34 (7.79)	0.026	0.093
		Control	−17.46 (7.32)		
LV	Basal	CoA	−19.15 (10.4)	0.355	0.639
		Control	−22.54 (11.5)		
LV	Medial	CoA	−11.77 (4.30)	0.636	0.818
		Control	−10.97 (6.17)		
RV	Apical	CoA	−15.90 (7.74)	0.808	0.850
		Control	−14.10 (6.22)		
RV	Basal	CoA	−16.79 (8.22)	0.850	0.850
		Control	−16.26 (8.03)		
RV	Medial	CoA	−16.35 (8.27)	0.441	0.662
		Control	−14.30 (6.48)		

SD—sample standard deviation, CoA—aortic coarctation, Inter V—interventricular septum; LV—left ventricle, RV—right ventricle.

**Table 4:** Intra-class-correlation coefficient and numerical results of Bland–Altman analysis.

Wall	Segment	Rater 1 Mean (SD)	Rater 2 Mean (SD)	*p*-Value *	ICC [95% CI]	Bias [95% CI]
LV	Basal (%)	−21.47 (11.15)	−21.10 (9.95)	0.725	0.80 [0.66, 0.89]	−0.37 [−2.47, 1.74]
	Medial (%)	−11.22 (5.61)	−11.28 (6.72)	0.943	0.65 [0.43, 0.79]	0.06 [−1.58, 1.70]
	Apical (%)	−15.52 (7.92)	−15.24 (8.04)	0.756	0.75 [0.57, 0.86]	−0.28 [−2.08, 1.52]
RV	Basal (%)	−16.43 (7.99)	−17.32 (9.05)	0.240	0.84 [0.73, 0.91]	0.89 [−0.62,2.40]
	Medial (%)	−14.95 (7.06)	−15.71 (7.18)	0.138	0.90 [0.82, 0.94]	0.76 [−0.26,1.78]
	Apical (%)	−14.29 (7.42)	−15.50 (7.15)	0.038	0.91 [0.83, 0.95]	0.86 [−0.07,1.80]
Inter V	Basal (%)	−12.58 (4.83)	−12.41 (4.96)	0.797	0.65 [0.43, 0.80]	−0.17 [−1.46, 1.13]
	Medial (%)	−17.48 (4.59)	−18.05 (4.38)	0.204	0.80 [0.65, 0.89]	0.58 [−0.33, 1.48]
	Apical (%)	−21.23 (8.43)	−23.84 (8.94)	0.002	0.83 [0.70, 0.90]	2.61 [0.99, 4.22]
LV	Global	−16.16 (4.15)	−16.56 (4.14)	0.297	0.83 [0.70, 0.90]	0.40 [−0.37, 1.18]
RV free wall	free wall (%)	−15.64 (7.18)	−16.50 (7.64)	0.102	0.90 [0.82, 0.95]	0.86 [−0.18, 1.89]
RV4C	4C (%)	−13.66 (5.06)	−13.87 (5.31)	0.574	0.90 [0.82, 0.94]	0.21 [−0.53, 0.97]

SD = sample standard deviation; * *p*-values obtained from paired Student’s *t*-test; ICC = intra-class correlation coefficient; bias = mean of differences; 95% CI = 95% confidence interval; Inter V—interventricular septum; LV—left ventricle, RV—right ventricle, RV4C—right ventricle four chambers.

## Data Availability

The raw data presented in this study can be obtained upon reasonable request addressed to Liliana Gozar (lili_gozar@yahoo.com).

## Supplementary Materials

The following are available online at https://www.mdpi.com/article/10.3390/jcm10194579/s1, Figure S1: Bland-Altman plot with the representation of the mean differences with 95% confidence interval (doted line and blue area), limits of agreement LOA for the speckle-tracking cardiac measurements on left ventricle, Figure S2: Bland-Altman plot with the representation of the mean differences with 95% confidence interval (doted line and blue area), limits of agreement LOA for speckle-tracking cardiac measurements on right ventricle., Figure S3: Bland-Altman plot with the representation of the mean differences with 95% confidence interval (doted line and blue area), limits of agreement LOA for speckle-tracking cardiac measurements on intraventricular.
