# Supplementary material for: Speckle-Tracking Global Longitudinal and Regional Strain Analysis in Neonates with Coarctation of Aorta: A Case-Control Study
Source: J Clin Med. 2021 Oct 2;10(19):4579. doi: 10.3390/jcm10194579 (PMC8509133; doi:10.3390/jcm10194579)
Supplement: Supplementary file 1 [file jcm-10-04579-s001.zip › jcm-1382005-supplementary.pdf]

Supplementary Materials

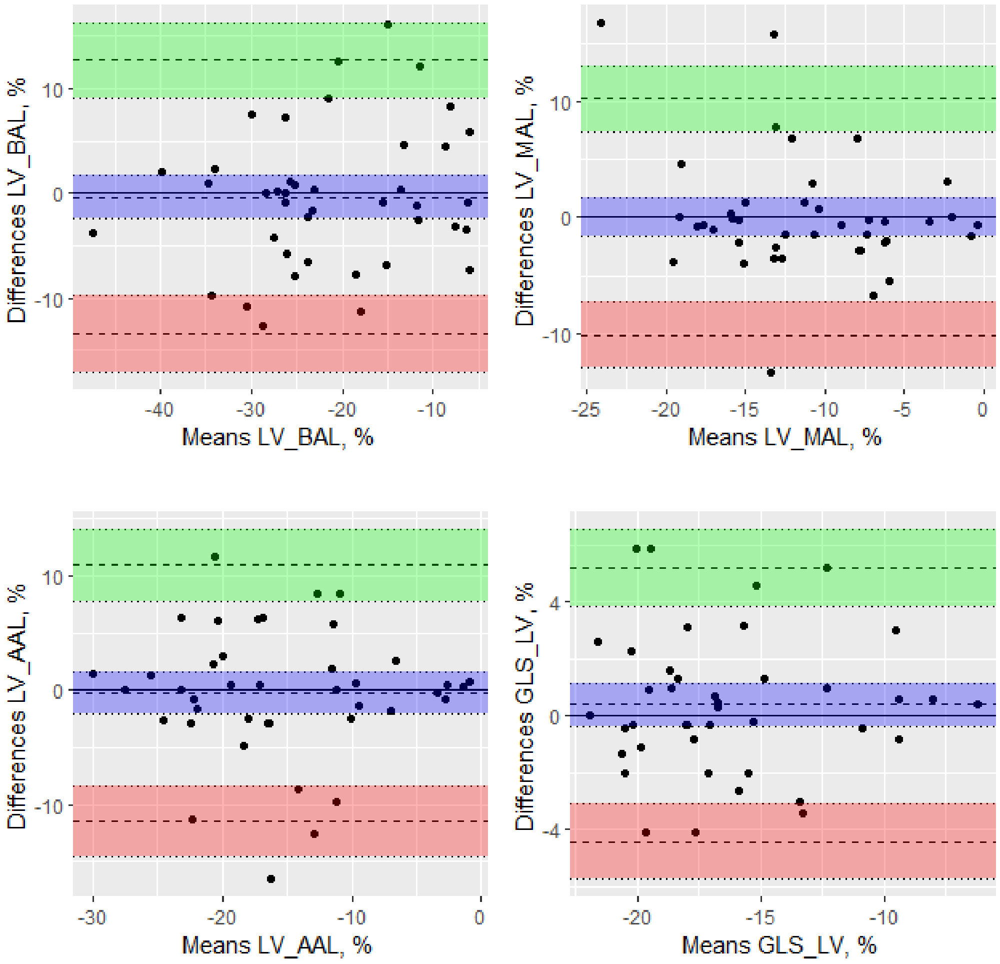

Figure S1. Bland-Altman plot with the representation of the mean differences with 95% confidence interval (doted line and blue area), limits of agreement LOA (Lower limit of agreement with 95% confidence interval were represented by doted line and green area while Upper limit of agreement with 95% confidence interval were represented by doted line and red area) for the speckle-tracking cardiac measurements on left ventricle. LV- left ventricle, BAL- basal segment, MAL- medial segment, AAL- apical segment, GLS- global longitudinal strain.

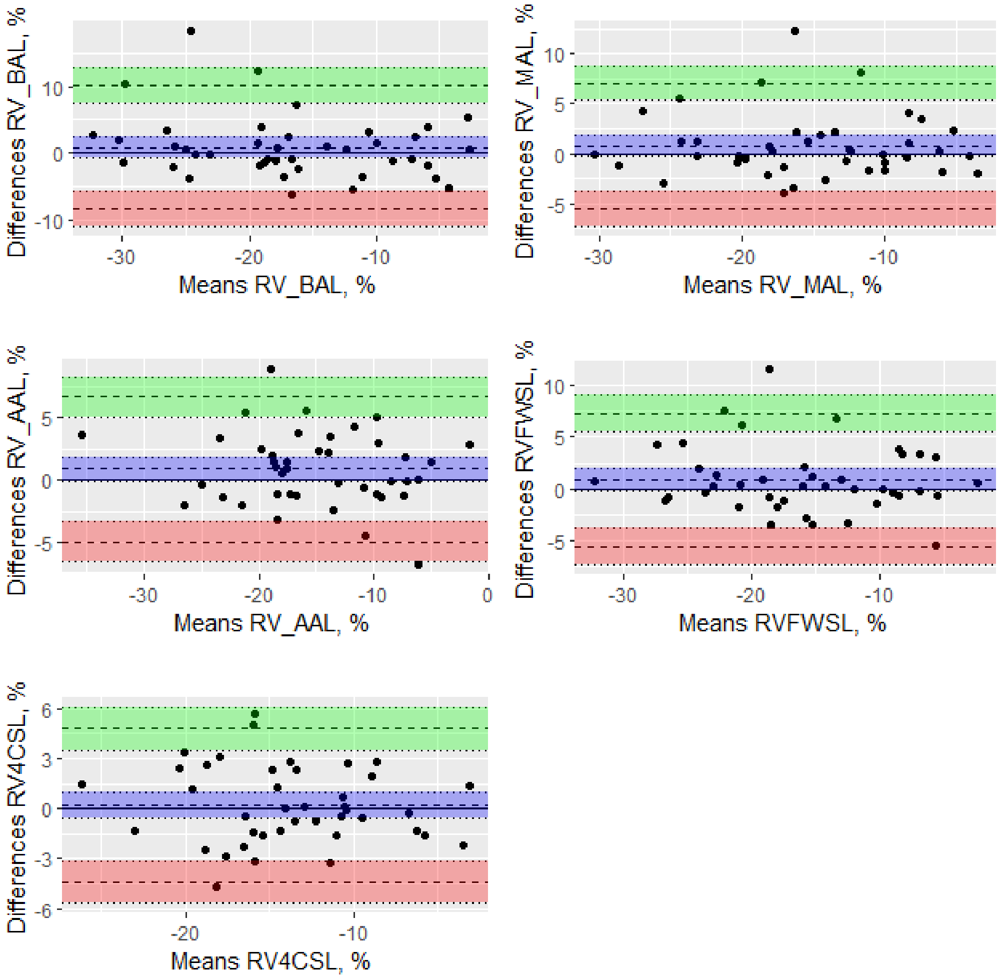

Figure S2. Bland-Altman plot with the representation of the mean differences with 95% confidence interval (doted line and blue area), limits of agreement LOA (Lower limit of agreement with 95% confidence interval were represented by doted line and green area while Upper limit of agreement with 95% confidence interval were represented by doted line and red area) for speckle-tracking cardiac measurements on right ventricle. RV- right ventricle, BAL- basal segment, MAL- medial segment, AAL- apical segment, RVFWSL – right ventricle longitudinal free wall strain, RV4CSL – right ventricle four-chamber strain.

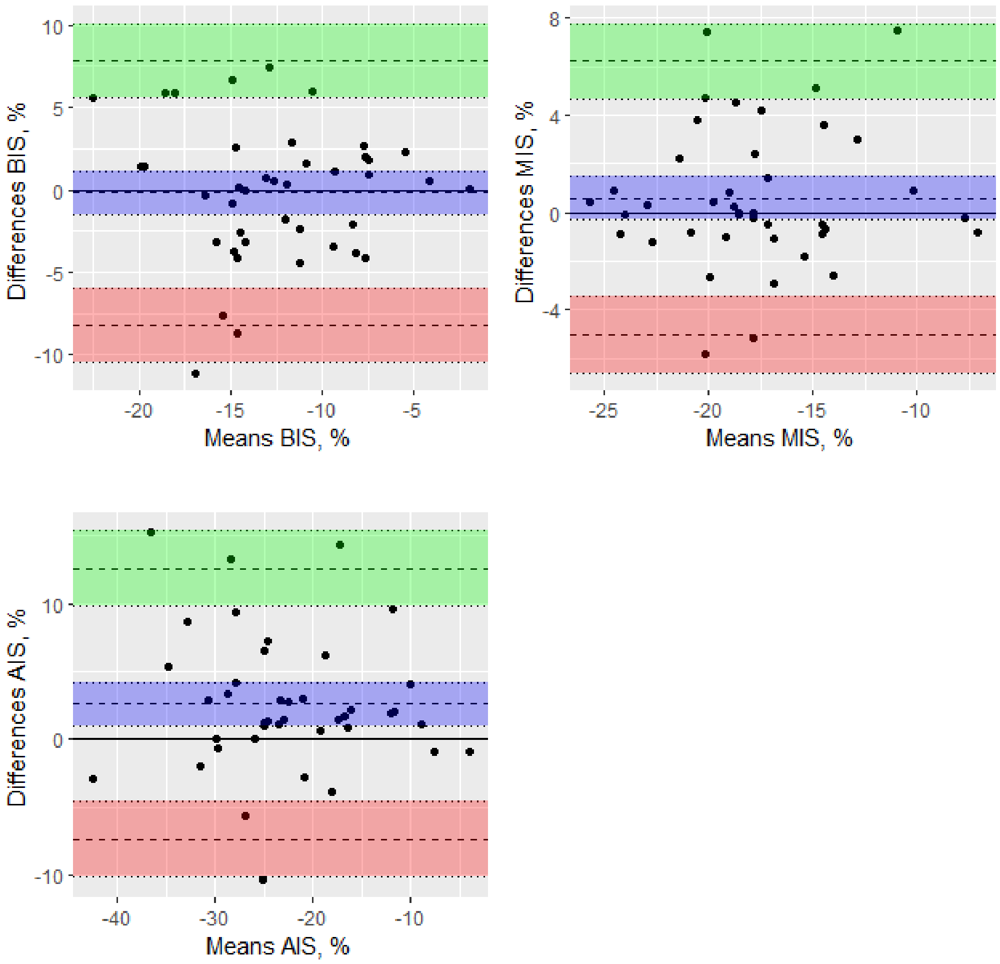

Figure S3. Bland-Altman plot with the representation of the mean differences with 95% confidence interval (doted line and blue area), limits of agreement LOA (Lower limit of agreement with 95% confidence interval were represented by doted line and green area while Upper limit of agreement with 95% confidence interval were represented by doted line and red area) for speckle-tracking cardiac measurements on interventricular septum. BIS- basal segment of interventricular septum, MIS – medial segment of interventricular septum, AIS – apical segment of interventricular septum.
